# Femoral Artery Infusion of αMSH Increases Muscle Thermogenesis and Promotes Glucose Uptake in Ovariectomized Ewes

**DOI:** 10.1210/endocr/bqae156

**Published:** 2024-11-19

**Authors:** Belinda A Henry, Michael A Cowley, Zane B Andrews, Iain J Clarke

**Affiliations:** Department of Physiology, Metabolism, Diabetes and Obesity Program, Monash Biomedicine Discovery Institute, Monash University, Clayton, VIC 3800, Australia; Department of Physiology, Metabolism, Diabetes and Obesity Program, Monash Biomedicine Discovery Institute, Monash University, Clayton, VIC 3800, Australia; Department of Physiology, Metabolism, Diabetes and Obesity Program, Monash Biomedicine Discovery Institute, Monash University, Clayton, VIC 3800, Australia; Department of Physiology, Monash University, Clayton, VIC 3800, Australia

**Keywords:** skeletal muscle, mitochondria, energy expenditure, melanocortins

## Abstract

The melanocortin system is fundamental to neural control of energy balance and long-term weight regulation. Recent evidence shows that melanocortins also act at peripheral tissues to regulate metabolism, independent of the brain or the sympathetic nervous system (SNS). One such target is skeletal muscle, which contributes to energy expenditure through changes in adaptive thermogenesis. We aimed to determine 1) whether direct femoral infusion of α-melanocyte–stimulating hormone (αMSH) could increase muscle heat production independent of SNS activation and 2) if αMSH-induced skeletal muscle heat production was associated with altered mitochondrial function. Dataloggers were implanted into one hind leg of ovariectomized ewes and set to record vastus lateralis temperature every 15 minutes. A cannula was inserted into one femoral artery for infusion of either αMSH (0.1 µg/h) or saline. Femoral infusion of αMSH increased (*P* < .0001) skeletal muscle heat production, without effect on food intake. State 4 respiration increased (*P* < .05) and the respiratory control ratio decreased (*P* < .05) in mitochondria isolated from αMSH-treated animals. In addition, femoral infusion of αMSH reduced plasma glucose concentration in the femoral, but not the jugular vein; there was no effect of αMSH treatment on nonesterified fatty acid concentrations. These data suggest that αMSH can act locally to increase glucose uptake. We further show that blockade of the α- and β-adrenergic limbs of the SNS with either phentolamine or propranolol infusion had no effect on αMSH-induced skeletal muscle heat production. Overall, we show that αMSH acts directly at skeletal muscle to promote glucose uptake and increase energy expenditure via mitochondrial thermogenesis.

Pro-opiomelanocortin (POMC) neurons within the arcuate nucleus of the hypothalamus integrate peripheral endocrine and metabolic signals to regulate energy balance and glucose homeostasis (1). The leptin-melanocortin nexus is essential to long-term weight control, whereby POMC is enzymatically cleaved to produce the melanocortin peptides (α-, β-, and γ-melanocyte–stimulating hormone [MSH]), which bind to the melanocortin 3 and 4 receptors (MC3R/MC4R) located within the central nervous system to control food intake and energy expenditure (2, 3). In humans, mutations in MC4R are the most common cause of genetic obesity (2, 4) and the use of setmelanotide, an MC4R agonist, is an effective pharmacotherapy for the treatment of monogenetic and syndromic obesity phenotypes (5, 6). In addition to weight control, early electrophysiological studies showed that POMC neurons are regulated by glucose (7) and more recent in vivo work has demonstrated that insulin action on POMC neurons modulates peripheral glucose metabolism via regulation of hepatic glucose production (8). Thus, it has been firmly established that the central melanocortin pathway is integral to the control of body weight and glucose metabolism.

In addition to neuronally derived melanocortin peptides, αMSH is synthesized and secreted from the melanotropes of the intermediate lobe of the pituitary gland in sheep (9) and rodents (10, 11) and also by the corticotropes of the anterior pituitary in humans (12). In sheep, αMSH circulates within the bloodstream, where it is secreted in a pulsatile manner (13) and in humans, circulating αMSH increases postprandially (14). There are 5 melanocortin receptor subtypes, which are abundant in peripheral tissues. In addition to MC3R and MC4R found in the brain, MC1R is expressed in skin and MC2R is located in adipose tissues and the adrenal gland (15-17). The MC5R is expressed in adipose tissue, liver, and skeletal muscle, with recent studies evoking a role for MC5R in the control of metabolism in multiple species including birds, rodents, and humans (14, 18-20). Furthermore, polymorphisms in the *MC5R* gene have been associated with an obese phenotype, including increased body mass index and fat mass, and a reduction in resting metabolic rate (21). Given the catabolic nature of skeletal muscle, it remains possible that peripherally derived melanocortin peptides regulate energy expenditure and metabolic function via direct MC5R-driven effects on muscle tissues.

With regard to energy expenditure, the melanocortin peptides are known to act within the brain to regulate adaptive thermogenesis in brown adipose tissue (BAT) and skeletal muscle (22, 23). In muscle, cellular thermogenesis occurs via futile calcium cycling and uncoupling protein 3 (UCP3)-induced mitochondrial uncoupling (24, 25). In contrast to BAT, relatively little is known about the mechanisms that regulate skeletal muscle thermogenesis. In rodents, sarcolipin uncouples the futile calcium pathway and is therefore an endogenous activator of muscle thermogenesis (26). On the other hand, in large mammals such as sheep, intracerebroventricular infusion of leptin increases muscle thermogenesis via increased state 4 respiration and a switch to uncoupled mitochondrial respiration (27, 28). In addition, we have shown previously that peripheral administration of αMSH increases skeletal muscle heat production (14), although the underpinning mechanisms have not been explored.

Given that muscle comprises approximately 40% of total body mass, small changes in metabolic flux and energy expenditure are likely to have substantial ramifications for long-term weight regulation and metabolic health. As mentioned earlier, linkage studies have shown that MC5R polymorphisms are associated with reduced energy expenditure in humans and our work suggests that peripheral administration of αMSH can increase muscle temperature. In light of this, we aimed to determine the effects of low-dose femoral artery infusion of αMSH on muscle temperature, mitochondrial respiration, and plasma glucose and nonesterified fatty acid (NEFA) concentrations. We further sought to determine whether αMSH-induced heat production is mediated centrally and therefore caused by increased activation of the sympathetic nervous system (SNS). We hypothesized that αMSH exerts a direct beneficial effect at skeletal muscle itself to improve metabolic function and enhance energy expenditure via thermogenic pathways.

## Methods and Materials

### Animals

Animal work was approved by the Monash Animal Research Platform Ethics Committee, in accordance with the 2004 Australian Code of Practice for the Care and Use of Animals and the Prevention of Cruelty to Animals Act 1966. All studies were carried out using ovariectomized Corriedale ewes. Animals were ovariectomized at least 1 month prior to experimentation to prevent the confounding effects of fluctuating ovarian steroids on temperature recordings. All experiments were carried out using a randomized crossover design in which sheep received both saline and αMSH treatments. Animals were of normal body weight (52.5 ± 1.3 kg) and were maintained on a “meal” feeding regimen, with lucerne chaff being available between 11:00 and 16:00 hours daily. We have previously demonstrated that temporal food restriction or a meal feeding program entrains a postprandial increase in skeletal muscle thermogenesis (25, 28).

### Surgical Procedures

Prior to all surgeries, animals received an intramuscular injection of the analgesic Rimadyl (Zoetis, Australia) (4 mg/kg body weight). Silastic cannula were inserted into the femoral artery and vein for infusion and blood sampling, respectively. To do this, animals were anesthetized via an intravenous injection of thiopentone sodium (20 mg/kg body weight); animals were then intubated for maintenance of anesthesia via inhalation of isoflurane (1.5%-2.5%). Animals were laid prone and an incision was made in the groin region. For cannulation of the femoral artery, silastic tubing was inserted into the superficial branch of the femoral artery and then threaded into the main artery (∼15 cm). The superficial branch was selected as this does not provide any blood supply to the muscle. A second silastic cannula was inserted into the femoral vein and both cannulae were tunneled under the skin and externalized on the flank of the animal. Cannulae were connected to 3-way taps and kept patent with heparinized (100 units/mL) saline. Cannulae were flushed 3 times per day with 20 mL of heparinized saline to ensure patency was maintained. In addition, temperature probes (Dataloggers, SubCue) were implanted into the skeletal muscle of the infusion limb, with the recording side facing the vastus lateralis muscle (25, 28). Dataloggers were set to record temperature at 15-minute intervals for the duration of the experimental period.

### Experiment 1. Effect of Direct Infusion of α-Melanocyte–Stimulating Hormone Into the Femoral Artery on Skeletal Muscle Heat Production

#### Part A

To examine the effect of αMSH on skeletal muscle heat production, a cannula was inserted into the femoral artery and vein and a datalogger was implanted as detailed earlier. Sheep were meal-fed for at least 1 week prior to the onset of experimentation, and food intake was measured daily.

One day prior to infusion, a cannula was inserted into one jugular vein to facilitate blood sampling; this was kept patent with heparinized saline. The day of treatment, animals received infusion of either heparinized (50 K; 110 µL/h) saline or 0.1 µg/h αMSH (Auspep) between 10:00 and 16:00 hours (n = 4/group). Blood samples (5 mL) were simultaneously collected both from the jugular and femoral veins every 30 minutes between 09:00 and 17:00 hours. Blood was collected into a heparinized tube, centrifuged at 3000 rpm for 10 minutes, and the plasma harvested then frozen and stored at −20 °C until analyzed. Glucose and NEFA concentrations were measured as previously described (28).

#### Part B

In addition to measuring muscle temperature, we examined the effect of αMSH infusion on mitochondrial function. Once the temperature responses to αMSH were established in part A of experiment 1, the infusions were repeated (as described earlier) and a muscle biopsy was collected (n = 4/group). The infusions were initiated at 10:00 hours, animals were fed at 11:00 hours, and the muscle biopsy collected at 12:00 hours. For tissue collection, animals were placed under short-term anesthesia (15 minutes) using intravenous thiopentone sodium (15 mL Thiobarb; Jurox). Muscle was collected under sterile conditions from the vastus lateralis muscle, adjacent to the datalogger. Mitochondria were immediately isolated by differential centrifugation, as previously described (27). Mitochondrial respiration was assessed in duplicate using a Clark-type electrode (Hansatech Instruments) at 37 °C with 250 µg of mitochondrial protein. Four key states of mitochondrial respiration were measured, including 1) substrate-driven respiration, 2) state 3 or coupled respiration (indicative of oxidative phosphorylation and adenosine triphosphate [ATP] synthesis), 3) state 4 or uncoupled respiration (adaptive thermogenesis), and 4) total respiratory capacity, which used a protonophore to disrupt the proton gradient. To assess substrate-drive respiration, mitochondria were stimulated with pyruvate and malate (5 and 2.5 mM) as oxidative substrates in respiration buffer (mannitol 230 mM, sucrose 70 mM, MgCl2 2 mM, K2HPO4 5 mM, and 0.1% bovine serum albumin). State 3 (coupled) respiration was assessed by the addition of adenosine diphosphate (150 µM) to drive ATP synthesis, which was subsequently inhibited by the ATP synthase inhibitor, oligomycin (1 µM), as an indicator of state 4 (uncoupled) respiration. The protonophore, carbonyl cyanide-p-trifluoromethoxyphenylhydrazone (FCCP) (1 µM), was added to disrupt the proton transport chain and thus measure maximal respiratory capacity. To determine the relative contribution of coupled vs uncoupled respiratory states, the respiratory control ratio (RCR) was calculated as state 3 respiration/state 4 respiration.

#### Data analyses

Longitudinal temperature data was analyzed using a 2-way analysis of variance (ANOVA) comparing overall temperature differences between saline- (control) and αMSH-treated groups. The effect of αMSH on food intake and measures of mitochondrial respiration were determined using an unpaired *t* test. The effects of αMSH on plasma concentrations of glucose and NEFA were analyzed using a 2-way ANOVA. In addition to assessing the longitudinal changes in plasma metabolite concentrations, the effect of αMSH infusion on the mean concentration of glucose and NEFA in samples collected from the femoral and jugular veins was determined using an unpaired *t* test. Statistical significance was set at *P* less than .05.

### Experiment 2. Role of α- and β-Adrenoceptors in α-Melanocyte–Stimulating Hormone–Induced Heat Production in Skeletal Muscle

#### Part A

To determine whether the effect of αMSH on skeletal muscle heat production was possibly mediated via activation of the SNS, we coinfused the nonspecific α or β adrenoceptor antagonists, phentolamine and propranolol, respectively. Double0infusion cannulas were inserted into the femoral artery as detailed earlier (experiment 1) and a datalogger was implanted into the hind limb to record temperature from the vastus lateralis muscle every 15 minutes. Animals (n = 5-6/group) were meal-fed between 11:00 and 16:00 hours daily. The day of infusion animals were randomly assigned to one of the following groups: 1) vehicle and saline, 2) vehicle and 0.1 µg/h αMSH, 3) phentolamine and saline, 4) phentolamine and αMSH, 5) propranolol and saline, or 6) propranolol and αMSH. Phentolamine and propranolol were dissolved in water at a concentration of 10 mg/mL. To initiate phentolamine and propranolol treatment, animals received a 20-mg bolus at 09:00 hours, after which the infusion was maintained at a rate of 10 mg/h. At 10:00 hours, saline or αMSH infusion was commenced. All infusions ceased at 16:00 hours.

To demonstrate the effectiveness of the dose of adrenergic antagonists, an additional experiment was carried out in ovariectomized ewes (n = 4/group) to demonstrate the effect of femoral artery infusion of propranolol on leptin-induced heat production in skeletal muscle. Intracerebroventricular infusion cannula were inserted into one lateral ventricle of the brain as previously described (28). A femoral artery cannular was inserted into one hind leg as described for experiment 1. A datalogger temperature probe was implanted into the skeletal muscle of one hind leg with the recording side adjacent to the vastus lateralis muscle. Propranolol was dissolved in water at a concentration of 10 mg/ml. Propranolol or vehicle were infused directly into the femoral artery. Leptin or vehicle (artificial cerebrospinal fluid: 150 mm NaCl, 1.2 mm CaCl, 1 mm MgCl, and 2.8 mm KCl) were infused into the lateral ventricle. At 09:00 animals received a 20-mg bolus of propranolol and immediately after the infusion was initiated at dose of 10 mg/h until 16:00 hours. Leptin (10 µg/h) or artificial cerebrospinal fluid was infused into the lateral ventricle between 10:00 and 16:00 hours.

#### Data analyses

To assess the effects of phentolamine and propranolol on αMSH-induced changes in skeletal muscle heat production, we calculated the change in peak temperature response and the area under the curve (AUC) temperature change. Statistical analyses were performed using a one-way ANOVA and post hoc comparisons were performed using a Sidak multiple comparison test. Statistical significance was set at *P* less than .05.

## Results

### Experiment 1. Effect of Direct Infusion of α-Melanocyte–Stimulating Hormone Into the Femoral Artery on Skeletal Muscle Heat Production

**Figure 1.**
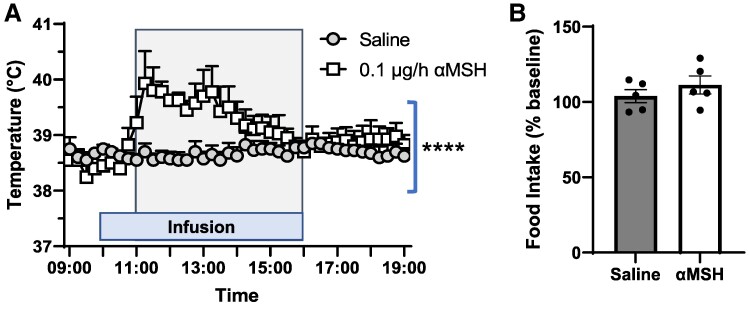
The effect of femoral artery infusion of α-melanocyte–stimulating hormone (αMSH) (0.1 µg/h) or saline on (A) skeletal muscle heat production and (B) food intake. Peripheral infusion of αMSH increased (****P < .0001) muscle temperature in the vastus lateralis muscle of the infusion limb. On the other hand, food intake was similar in saline- and αMSH-treated animals. All data are presented as the mean ± SEM.

Direct low-dose (0.1 µg) infusion of αMSH into the femoral artery caused a substantial increase (*P* < .0001) in skeletal muscle temperature in ovariectomized ewes (Fig. 1A). Despite the increase in skeletal muscle heat production, there was no effect of peripheral αMSH treatment on food intake (Fig. 1B).

**Figure 2.**
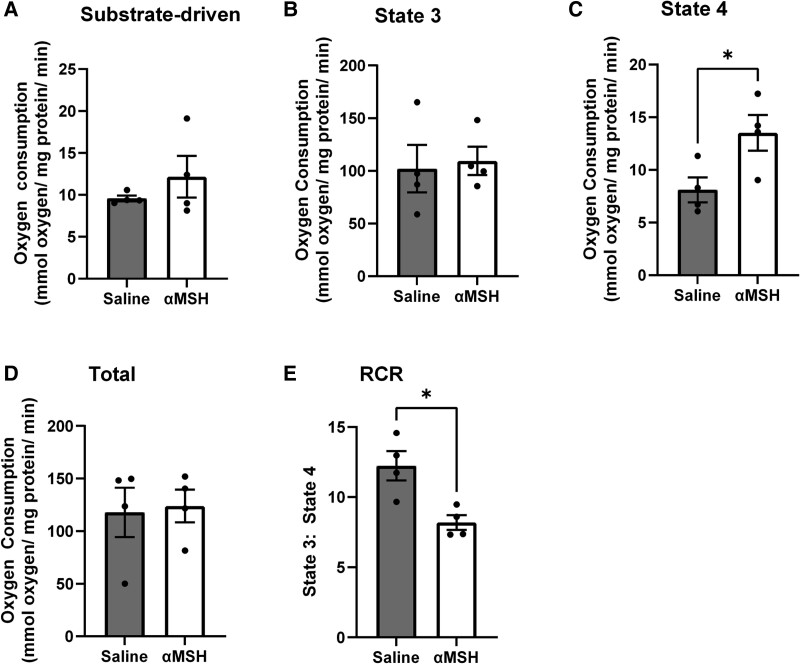
The effect of femoral artery infusion of α-melanocyte–stimulating hormone (αMSH) (0.1 µg/h) or saline on stages of cellular respiration in mitochondria isolated from the vastus lateralis muscle of the infusion limb. To assess mitochondrial respiration, we measured (A) substrate-driven respiration; (B) state 3 respiration; (C) state 4 respiration; (D) total respiratory capacity; and (E) the respiratory control ration (RCR). Substrate-driven and state 3 respiration as well as the total respiratory capacity was similar in αMSH- and saline-treated groups. In contrast, state 4 respiration was increased (*P < .05) and the RCR was reduced (*P < .05) by αMSH treatment. All data are presented as the mean ± SEM. Statistical analyses was performed using an unpaired *t* test.

To ascertain whether the changes in muscle temperature were associated with altered subcellular functions, we isolated mitochondria from the muscle of saline- and αMSH-treated animals and measured mitochondrial function. To do this, we measured substrate (pyruvate and malate)-driven (Fig. 2A), state 3 (Fig. 2B) and state 4 (Fig. 2C), total respiratory capacity (FCCP-driven respiration; Fig. 2D), and the respiratory control ratio (state 3: state 4 respiration; Fig. 2E). There was no effect of αMSH infusion on substrate-driven respiration (see Fig. 2A), state 3 (coupled) respiration (see Fig. 2B), or the maximal respiratory capacity (see Fig. 2D). On the other hand, state 4 (uncoupled) respiration (see Fig. 2C) was increased (*P* < .05) and the respiratory control ratio (state 3: state 4 respiration; see Fig. 2E) was reduced by αMSH treatment, indicating a switch toward uncoupled respiration, a hallmark of adaptive thermogenesis.

**Figure 3.**
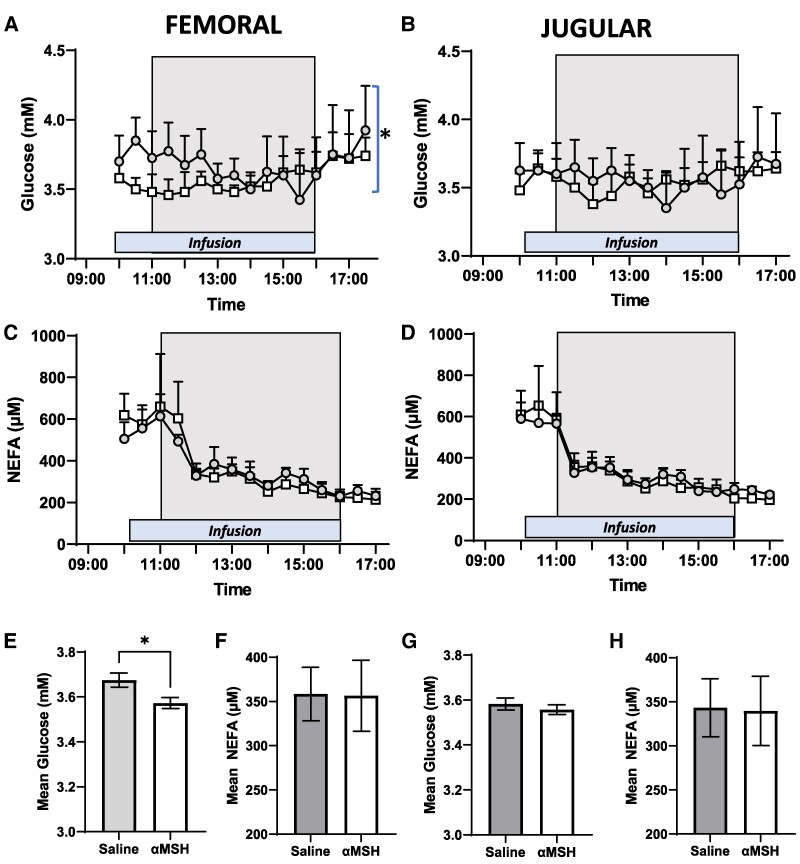
Serial measurement of plasma glucose and nonesterified fatty acids (NEFA) from the femoral and jugular vein in saline (circles) and α-melanocyte–stimulating hormone (αMSH)-treated (squares) animals. (A-D) The box depicts the feeding window and the bar depicts the period of infusion. In the femoral vein, infusion of αMSH (0.1 μg/h) decreased (*P < .05) A, the plasma concentration of glucose, although (B) there was no effect of αMSH treatment on glucose concentration in the jugular vein. With regard to NEFA, the onset of feeding reduced concentrations both in the (C) femoral and (D) jugular vein, but there was no effect of αMSH treatment. (E) The overall mean concentration of glucose in the femoral vein was lower (*P < .05) in αMSH-treated animals compared to their control counterparts. In contrast, the mean concentration of (G) glucose in the jugular vein and NEFA concentration both in the (F) femoral and (H) jugular veins were similar between αMSH- and saline-treated groups. All data are presented as the mean ± SEM.

In addition to measuring food intake and physiological/cellular indices of skeletal muscle thermogenesis, we assessed the effect of femoral artery infusion of αMSH on the concentration of both glucose and NEFAs in samples taken from the jugular and femoral (infusion side) veins. There was an initial effect of αMSH to cause localized reduction (*P* < .05) in plasma glucose concentrations in the femoral vein (Fig. 3A). In contrast, there was no effect of αMSH treatment on peripheral glucose concentrations in the jugular vein. Overall, the mean concentration of glucose across the entire sampling period was lower (*P* < .05) in the femoral vein of αMSH-treated animals (Fig. 3E) but there was no effect of αMSH on glucose concentrations in the jugular vein (Fig. 3F). As shown previously (28), NEFA levels were reduced in the femoral (Fig. 3C) and jugular vein samples in response to feeding (Fig. 3D), but we now demonstrate that there was no effect of αMSH treatment. The mean concentrations of NEFA both in the femoral (Fig. 3G) and jugular (Fig. 3H) veins were similar in αMSH-treated and control animals across the sampling period.

### Experiment 2. Role of α- and β-Adrenoceptors in α-Melanocyte–Stimulating Hormone–Induced Heat Production in Skeletal Muscle

**Figure 4.**
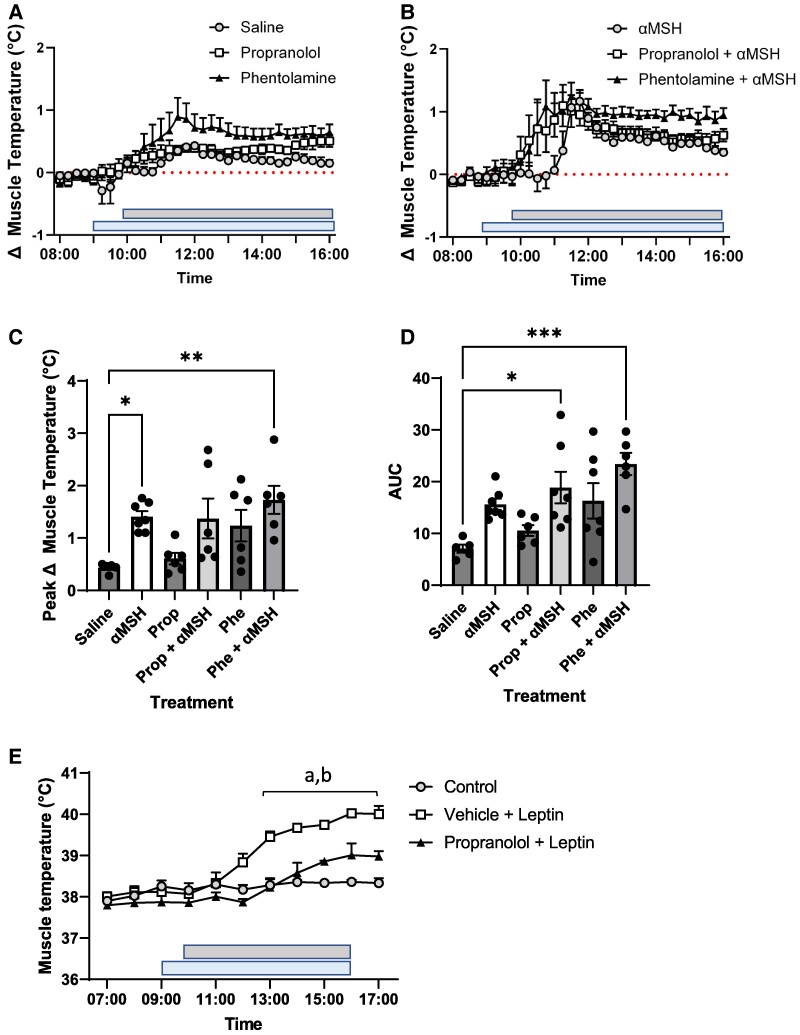
Effect of phentolamine and propranolol treatment on α-melanocyte–stimulating hormone (αMSH)-induced skeletal muscle heat production. There was no statistically significant effect of phentolamine or propranolol treatment on the longitudinal muscle temperature pattern in either (A) saline- or (B) αMSH-treated animals. The peak temperature response was greater in αMSH alone (*P < .05) and αMSH-phentolamine-treated (**P < .01) animals compared to the (C) saline controls. With regard to the area under the curve (AUC), this was greater both in αMSH-phentolamine (*P < .05) and αMSH-propranolol (***P < .001) compared to the (D) saline-treated controls. Notably, the peak temperature response or AUC in αMSH-treated animals was not altered by coadministration of either (C) phentolamine or (D) propranolol. (E) In contrast, β-blockade with propranolol attenuated the leptin-induced skeletal muscle heat production in female sheep. (E) Skeletal muscle temperature increased by leptin treatment (^a^P < .05, control vs leptin groups). (E) Furthermore, propranolol infusion mitigated (^b^P < .05 leptin, vs leptin/propranolol) the ability of leptin to increase heat production in skeletal muscle.

To determine whether the effect of αMSH on skeletal muscle temperature was via altered activity of the SNS, we examined the effects of α- and β-adrenergic antagonists on αMSH-induced heat production. There was no effect of phentolamine (Fig. 4A) or propranolol (Fig. 4B) administration on αMSH-induced longitudinal changes in skeletal muscle temperature. Peak temperature increases in skeletal muscle temperature were greater (*P* < .05) in αMSH-treated animals compared to the control group, irrespective of propranolol treatment (Fig. 4C). The temperature response in terms of AUC was greater in the αMSH-phentolamine and αMSH-propranolol groups compared to the saline control (Fig. 4D). Importantly, the peak temperature response and the AUC were similar in the αMSH-vehicle treated group compared to both the αMSH-phentolamine– and αMSH-propranolol–treated animals (see Fig. 4C and 4D). Thus, administration of either phentolamine or propranolol did not diminish the effectiveness of αMSH to increase muscle heat production.

To demonstrate that the dosage of the adrenergic antagonists was functionally relevant, we performed an auxiliary study ascertaining the effect of propranolol administration on leptin-induced skeletal muscle heat production. Our previous work has shown that leptin acts at the brain to increase skeletal muscle thermogenesis (27, 28). Herewith, we demonstrate that β-adrenoceptor blockade by femoral artery infusion of propranolol reduces (*P* < .01) leptin-induced heat production in skeletal muscle (Fig. 4E). This observation supports the notion that leptin acts at the brain to increase thermogenesis via increased sympathetic drive to skeletal muscle.

## Discussion

The present study provides evidence that femoral infusion of αMSH acts directly on skeletal muscle to increase heat production via cellular thermogenesis in ovariectomized ewes. We show that the effect of αMSH is via direct action on skeletal muscle since 1) femoral infusion of low-dose αMSH had no concomitant effect on food intake, 2) coadministration of either α- or β- adrenergic antagonists, phentolamine or propranolol, did not affect the αMSH-induced temperature increase, and 3) αMSH infusion increased state 4 respiration and caused a preferential switch toward uncoupled respiration in isolated mitochondria. These data illustrate that the effect of αMSH to increase skeletal muscle heat production is not mediated via central action within the brain, nor is the effect through increased activity of the SNS. In addition, femoral infusion of αMSH caused a transient decrease in glucose concentration within the femoral vein, but not the jugular vein, indicative of an increase in localized (skeletal muscle) glucose uptake. Previous work in mice has shown that αMSH acts via MC5R in skeletal muscle to enhance glucose uptake (14). This suggests that the metabolic effect of αMSH to enhance glucose uptake in skeletal muscle is conserved across species. Thus, this study provides comparative evidence to demonstrate that αMSH acts directly at skeletal muscle to regulate energy expenditure and metabolic function in large mammals such as sheep.

In skeletal muscle, 2 key subcellular pathways have been linked to adaptive thermogenesis: futile calcium cycling and mitochondrial uncoupling. Futile calcium cycling occurs across the sarcoendoplasmic reticulum (SR), where activation of the sarcoendoplasmic ATPase (SERCA) 1 and 2a maintain calcium homeostasis by pumping Ca^2+^ into the SR; SERCA action requires hydrolysis of ATP, leading to cellular heat production (29, 30). On the other hand, in skeletal muscle of large mammals such as sheep, adaptive thermogenesis also occurs in mitochondria via UCP3 (30). In skeletal muscle of ovariectomized ewes, increased state 4 respiration in isolated mitochondria coincides with both postprandial and leptin-induced thermogenesis (25, 27). We now demonstrate that femoral artery infusion of αMSH increases skeletal muscle temperatures and elicits a concurrent increase in state 4 respiration in isolated mitochondria. Thus, peripherally derived αMSH can exert a physiological effect to increase energy expenditure through uncoupled respiration and the induction of adaptive thermogenesis, specifically in muscle.

Peripherally administered αMSH has previously been shown to reduce body weight in POMC knockout mice (31). This weight loss effect was attributed to peripheral action, since there was no effect of αMSH treatment on food intake in POMC-null female mice (31). It is well known that the satiety effect of the melanocortin peptides is mediated via the MC4R-expressing neurons in the paraventricular nucleus of the hypothalamus (32, 33). In the present study, femoral infusion of αMSH had no effect on satiety. In sheep, fluctuations in NEFAs reflect changes in temporal feeding patterns and, consistent with this, we found that concentrations of NEFAs were reduced after the onset of feeding, but there was no additional effect of αMSH (28). The lack of effect of αMSH on food intake substantiates the notion that peripheral melanocortin peptides can exert beneficial effects on body weight and energy balance via direct action at metabolic tissues, including skeletal muscle.

To further solidify the concept that the effect of αMSH on skeletal muscle thermogenesis is independent of central action on MC4R, we performed a series of antagonist studies using phentolamine and propranolol. In BAT, adaptive thermogenesis is principally under the control of the SNS, where genetic ablation of all 3 β-adrenoceptors on adipocytes renders BAT inert (34). Leptin acts at neurons located in the arcuate nucleus and the dorsomedial hypothalamus to stimulate sympathetic nerve activity, increasing thermogenesis in BAT in rodents (35-37). In female sheep, intracerebroventricular infusion of leptin increases state 4 uncoupled respiration in mitochondria, leading to enhanced skeletal muscle heat production (27, 28). Herein, we demonstrate that this increase in muscle temperature can be abolished by direct femoral infusion of the nonspecific β-adrenergic antagonist, propranolol, indicating that the SNS underpins the central effect of leptin on muscle thermogenesis. In muscle, β2 adrenoceptors are the predominant adrenergic receptor subtype and have been shown to be important regulators of blood flow, tissue anabolism via increased protein synthesis and reduced protein degradation, and energy balance (38, 39). In young healthy men, treatment with clenbuterol, a β2 adrenergic receptor agonist, increases resting energy expenditure as well as fatty acid oxidation (39). The present data indicate that leptin-induced increases in energy expenditure are due, in part, to sympathetic activation of thermogenic pathways in skeletal muscle.

In contrast, there was no effect of equivalent phentolamine or propranolol treatment on αMSH-induced heat production; this provides strong indication that the effect of αMSH on muscle thermogenesis was through direct action at the target tissue and was not indirect via the SNS. In rodents, numerous studies have consistently demonstrated a fundamental role for the SNS in mediating the effects of brain-derived melanocortins on BAT thermogenesis (40, 41). Central administration of either melanotan II (MC3/4 R agonist) or αMSH increases sympathetic drive and subsequent BAT temperature (40, 41). Furthermore, genetic deletion of the MC4R in mice leads to impaired diet- and cold-induced activation of BAT, as shown by the attenuated increase in UCP1 expression (42); this is indicative of reduced sympathetic responsiveness (43). Despite this, more recent studies suggest that αMSH may act directly on white adipose tissue to cause “browning.” In vitro studies in 3T3-L1 adipocytes show that αMSH treatment increases expression of *Ucp1* and other thermogenic genes, which coincided with an increase in uncoupled respiration in 3T3-L1 cells (44). Moreover, daily intraperitoneal injection of αMSH for 2 weeks increases the recruitment of UCP1-expressing brown adipocytes within the inguinal adipose tissue (44). This thermogenic morphology increased uncoupled respiration ex vivo and weight loss without an associated effect on satiety or food intake (44). These observations are consistent with the present data that demonstrate that peripheral administration of αMSH increases mitochondrial uncoupling or state 4 respiration and skeletal muscle thermogenesis without an associated effect on food intake. This highlights a novel pathway, independent of central action or the SNS, whereby melanocortin peptides regulate energy expenditure and therefore body weight.

In addition, αMSH can act on peripheral tissues to regulate both fatty acid and glucose metabolism. Previous in vitro studies have demonstrated that αMSH treatment increases glucose uptake in L6 myotubes and murine muscle explants (45). Further physiological studies show that peripheral αMSH secretion is increased in response to a glucose load in mice and that αMSH treatment increases glucose uptake in skeletal muscle via the MC5R (14). Similarly, αMSH-induced “browning” of inguinal adipose tissue is associated with increased glucose uptake (44). In the present study, we found that femoral infusion of αMSH reduced glucose levels in the femoral vein but not the jugular vein. This suggests that femoral infusion of αMSH had a localized effect to increase muscle glucose uptake.

The lack of an overall effect on plasma glucose concentration in samples from the jugular vein is likely due to the fact that sheep, being ruminants, absorb very little glucose directly from the diet, and whole-body glucose excursions are minimized via active gluconeogenesis and glucose-sparing pathways (46). Nonetheless, the present data suggest that in sheep, similar to rodents (14), αMSH promotes glucose uptake in skeletal muscle. In mice, peripheral infusion of αMSH improves glucose clearance during both glucose tolerance testing and hyperinsulinemic euglycemic studies, glucose clearance attenuated through either antibody neutralization or genetic deletion of the MC5R (14). Furthermore, the change in glucose clearance was thought to be due to increased glucose uptake since peripheral αMSH treatment had no effect on hepatic glucose production (14). Further investigation is required to conclusively show that αMSH increases glucose uptake in the sheep and that this has long-term beneficial effects on whole-body glucose metabolism. Nonetheless, the present study provides initial evidence that αMSH can act at peripheral target tissues to promote glucose uptake not only in mice, but also in ruminant species.

In conclusion, we used a large animal model to demonstrate that femoral infusion of αMSH increases skeletal muscle heat production independent of activation of the SNS. We showed that the increase in heat production is associated with elevated state 4 respiration in isolated mitochondria and reduced plasma levels of glucose in the femoral vein, indicative of enhanced localized glucose uptake. Further work is required to elucidate the molecular mechanisms underpinning the beneficial effects of αMSH on thermogenesis and glucose metabolism in skeletal muscle of sheep. Nonetheless, the present data emphasize that melanocortin peptides exert both central and peripheral effects to elicit advantageous effects on weight regulation and overall metabolic health.

## Data Availability

Some or all data sets generated during and/or analyzed during this study are not publicly available but are available from the corresponding author on reasonable request.

## Abbreviations

αMSH: α-melanocyte–stimulating hormone
ANOVA: analysis of variance
ATP: adenosine triphosphate
AUC: area under the curve
BAT: brown adipose tissue
MC5R: melanocortin 5 receptor
NEFA: nonesterified fatty acid
POMC: pro-opiomelanocortin
RCR: respiratory control ratio
SNS: sympathetic nervous system
SR: sarcoendoplasmic reticulum
UCP: uncoupling protein
